# Novel missense mutation in ligand binding domain of AR gene identified in patient with androgen insensitivity syndrome from Ukraine

**DOI:** 10.1002/ccr3.3566

**Published:** 2020-11-29

**Authors:** Dmytro Sirokha, Olexandra Gorodna, Dmytro Lozhko, Ganna Livshyts, Nataliya Zelinska, Liudmyla Livshits

**Affiliations:** ^1^ Institute of Molecular Biology and Genetics National Academy of Sciences of Ukraine (IMBG) Kyiv Ukraine; ^2^ Ukrainian Scientific and Practical Center for Endocrine Surgery, Transplantation of Endocrine Organs and Tissues Ministry of Health of Ukraine Kyiv Ukraine

**Keywords:** androgen insensitivity syndrome, androgen receptor, bioinformatics, ligand binding domain, pathogenic mutation

## Abstract

To improve diagnostic informativity of AR gene mutation analysis in patients with AIS, we recommend to include novel identified missense mutation c.2507T>G in the list of AIS‐causing mutations.

## Graphical abstract

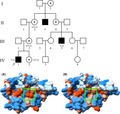

## 1 INTRODUCTION

In our study, we analyzed the pathogenicity of mutations detected in AR‐LBD in Ukrainian patients with different clinical AIS phenotypes and described the novel missense mutation c.2507T>G in patient with compete androgen insensitivity syndrome (CAIS). Pathogenicity of detected mutations was analyzed using bioinformatic tools and 3D in silico modeling.

Androgen insensitivity syndrome (AIS) is a disorder of sex development (DSD) with genetic etiology that occurs at a frequency of 1 in 20 000 live births and is the most common DSD in people with karyotype 46,XY.^1^ The phenotypes range from normal female genitalia in patients with complete androgen insensitivity syndrome (CAIS) to a wide range of ambiguous, undervirilized genitalia in patients with partial androgen insensitivity syndrome (PAIS).^2^ Mutations in the androgen receptor (AR) gene are found in majority individuals with CAIS and in a small number of individuals with PAIS. The AR gene encodes the androgen receptor, which is a member of the nuclear receptor superfamily of ligand‐dependent transcription factors, which also includes estrogen, progesterone, mineralocorticoid, and glucocorticoid receptors.^3^ The main function of the androgen receptor is the direct regulation of transcription. Binding of androgens to AR leads to a conformational change, allowing the transfer of the receptor from the cytoplasm to the nucleus, where it functions as a homodimer.^4^ Then, AR binds to a specific DNA sequence known as androgen response element (ARE) and interacts with other proteins in the nucleus to modulate transcriptional regulation.^5^ It was shown that androgen receptor undergoes phosphorylation, the patterns of which change after ligand binding. Phosphorylation of a certain amino acid residues has been found to affect various functions of the androgen receptor, such as activation of MAPK signaling cascade.^6^ ^,^ ^7^ ^,^ ^8^ Currently, more than 1000 mutations associated with AIS and prostate cancer have been described in the AR gene. Of those, about 600 were found in AIS patients, while 400 mutations fall in ligand binding domain (LBD) of AR protein.^9^ The aim of our study was to research the spectrum and pathogenicity of mutations in 6‐8 exons of the androgen receptor gene among patients with androgen insensitivity syndrome from Ukraine.

## 2 MATERIALS AND METHODS

### 2.1 Patients

Four patients with clinical features of AIS and available relatives from four unrelated Ukrainian families were investigated. Serum levels of testosterone (T), luteinizing hormone (LH), and follicle‐stimulating hormone (FSH) were quantified by electrochemiluminescence immunoassay (ECLIA) technology on Cobas e 411 (Roche Diagnostics, Switzerland). Elecsys Testosterone II, Elecsys LH, and Elecsys FSH kits were used according to the manufacturer's instructions.

### 2.2 Genetic analysis

Cytogenetic studies were performed on peripheral blood lymphocytes using Microscope Nikon Eclipse Ci, Software: Lucia Karyotyping and FISH Software according to standard protocols of chromosomal analysis (GTG‐banding, FISH—probes CEP, LSI (Probes: Yp11.3—SRY; Yp11.1‐q11.1—DYZ3; Yq12—DYZ1; CEP—DXZ1), Abbott Molecular, USA). DNA from peripheral blood lymphocytes was extracted by a hydrolysis of cell lysates with proteinase K followed by phenol extraction. The quality of DNA in samples was measured spectrophotometrically using ND‐1000 Spectrophotometer (NanoDrop). PCR was performed using 5xHOT FIREPol^®^ Blend Master Mix (Solis BioDyne, Estonia) according to the manufacturer's instructions, with primers described previously.^10^ Visualization was performed on a 2% agarose gel, fragments of target length were excised from agarose, and the PCR product was isolated and purified using Silica Bead DNA Gel Extraction Kit (Thermo Fisher Scientific) according to the manufacturer's instructions. Listed primers were further used for Sanger sequencing. Sequencing was performed using BigDye^®^ Terminator Kits (Thermo Fisher Scientific) on 3130 Genetic Analyzer (Applied Biosystems, Thermo Fisher Scientific).

### 2.3 Bioinformatics analysis and molecular modeling

Chromatograms were analyzed and converted from.ab1 format to.fasta sequence using open source SnapGene 4.3.11 software (GSL Biotech LLC). The sequences were aligned using the Nucleotide Blast (https://blast.ncbi.nlm.nih.gov/Blast.cgi), against reference sequences of 6, 7, 8 exons of AR gene (GenBank database under accession number NM_000044.6) provided by Ensembl Genome Browser database (https://www.ensembl.org/index.html). Genome assembly—GRCh38, transcript—ENST00000374690.9. The frequency of found SNPs was determined using gnomAD v2.1.1 (https://gnomad.broadinstitute.org/). Impact of the mutation was assessed using the: Variant Effect Predictor (https://www.ensembl.org/info/docs/tools/vep/index.html), Varsome (https://varsome.com/), and Human Splicing Finder (http://www.umd.be/HSF/).^11^ The probability of amino acid residue phosphorylation was verified using NetPhorest 2.1 (http://www.netphorest.info/), Group‐based Prediction System 5.0 (http://gps.biocuckoo.cn/), and PhosphoPICK (http://bioinf. scmb.uq.edu.au/phosphopick/phosphopick). To determine the potential pathogenic impact of polymorphisms on the protein structure, SIFT (Sorting Intolerant from Tolerant, an algorithm that predicts impact of mutation, based on amino acid conservancy), PolyPhen (based on structural and comparative evolutionary considerations), and MutationTaster (based on comparison with the integrated databases and tests according to the gene alteration they are causing) values were used.^12^ ^,^ ^13^ ^,^ ^14^ Three‐dimensional coordinates of the protein structures and information concerning interactions with ligands were obtained from the RSCB PDB database (http://www.rcsb.org/). Modeling of mutant proteins based on available 3D structures was performed using UCSF Chimera 1.14rc open source software.^15^ The protein stability change caused by single‐point mutation was calculated using Web server STRUM.^16^

## 3 RESULTS

Single‐nucleotide substitutions were detected in exons 7 and 8: three substitutions in exon 7 and one substitution in exon 8. Family history was positive in three cases and negative in one case. The 46,XY SRY‐positive karyotype was identified in all investigated patients. The presence of SRY sequence was confirmed by FISH with Yp11.3—SRY probe.

### 3.1 Patient 1

**Figure 1:**
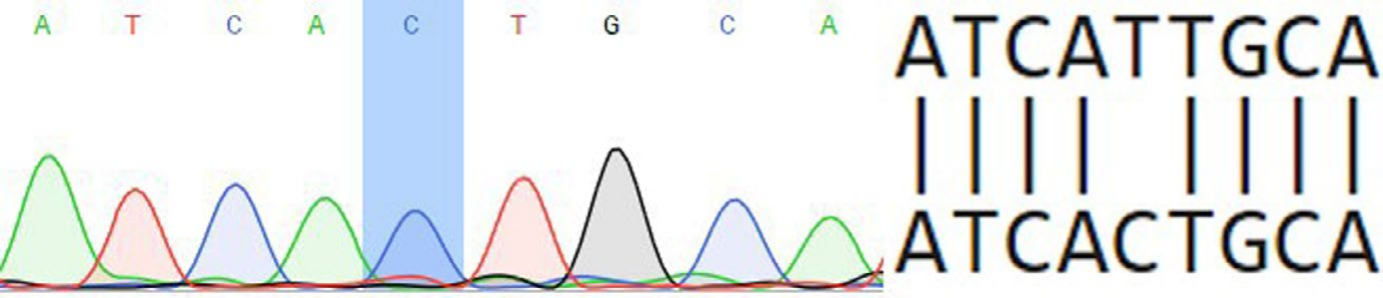
Partial electropherogram of exon 7 of androgen receptor (AR) gene

46,XY SRY + was born with ambiguous genitalia: urogenital sinus, micropenis, both testicles are located in the split scrotum, and the length of the blind‐ended vagina is 1.5 cm. Patient was registered in a female social sex. Laboratory tests: FSH 2.11 IU/L (0.2‐2.8), LH 4.91 IU/L (0.1‐1.3), total testosterone (TT) 2.15 ng/dL (<7‐20), and free testosterone (FT) 2.71 ng/mL (<4‐11). The clinical phenotype of this patient was determined as PAIS. The family history of PAIS was confirmed. Pedigree anamnesis showed that proband's sibs also have PAIS. Patient 1 was diagnosed with PAIS at the age of 1 year and 6 months. Mutation c.2528T>C (rs9332970) was identified (Figure 1). Detected substitution (missense mutation Ile843Thr) is located in exon 7. No allele frequency data are available. The pathogenicity of detected variant was estimated using the following scores:

- SIFT—0.001, damaging
- PolyPhen—0.998, probably damaging
- MutationTaster—0.9999, disease causing.

Based on listed results, we have predicted this variant as pathogenic.

### 3.2 Patient 2

**Figure 2:**
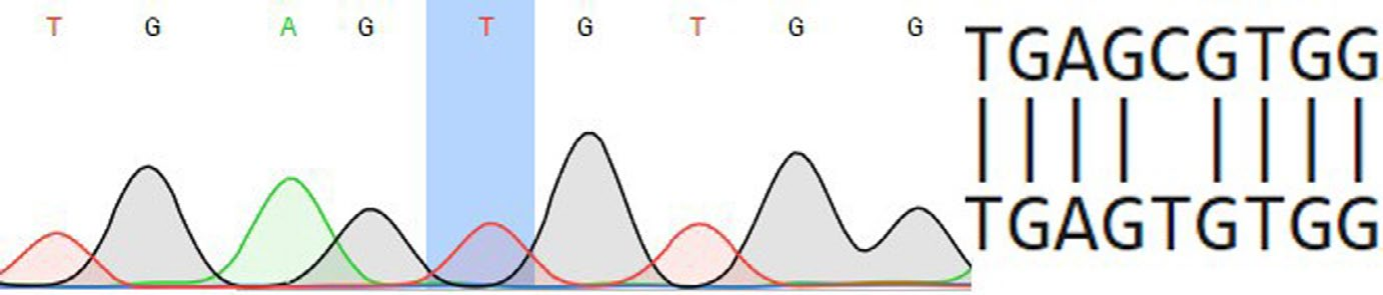
Partial electropherogram of exon 8 of androgen receptor (AR) gene

46,XY SRY + was born with ambiguous genitalia: micropenis, glandular hypospadias, right testicle is located in the inguinal canal, and left testicle is located in the hypoplastic scrotum. Patient was registered in a female social sex. The clinical phenotype of this patient was determined as PAIS, and the diagnosis was made at birth. We determined family history as negative using available pedigree data. Laboratory tests: FSH 159.9 IU/L (0.2‐2.8), LH 2.7 IU/L (0.1‐1.3), T 0.0002 ng/dL (<0.04‐0.11). Substitution c.2667C>T (rs137852594) was identified. This transversion is a samesense mutation (Ser889=) (Figure 2). Pathogenicity of silent mutation may be explained by the introduction of a new enhancer motif for the SRp55 spliceosome protein. Consequently, a new donor splice site appears at the end of the last exon 8, which causes the 3′UTR region translation, as predicted by Human Splicing Finder. Upon activation of the cryptic splice site, the wild‐type exon loses 67 nucleotides, and the protein becomes shorter by 25 amino acid residues (the last eight amino acids are not native). Thus, the resulting protein lacks fragment downstream of Met895. Particularly, the protein loses Ile899, which normally forms a binding pocket of the ligand binding domain. This directly affects the ability to bind the ligand dihydrotestosterone (DHT) and causes a loss of function.

### 3.3 Patient 3

**Figure 3:**
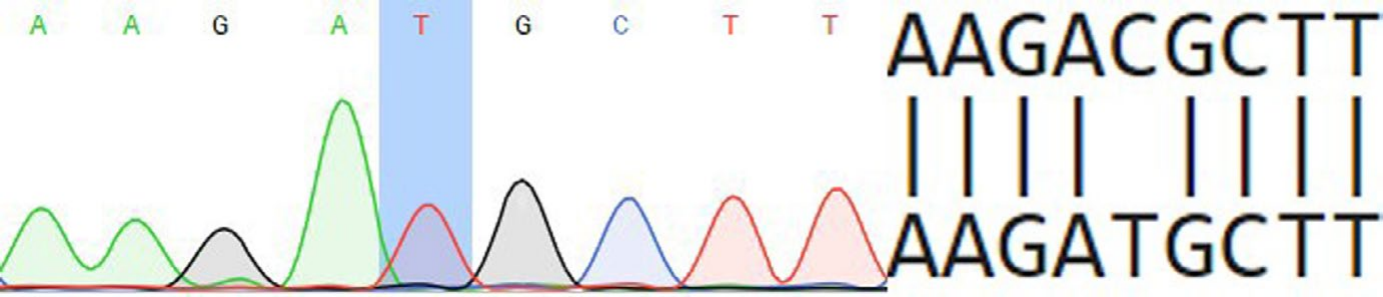
Partial electropherogram of exon 7 of androgen receptor (AR) gene

46,XY, SRY+, has started an examination at the age of 15 years for primary amenorrhea and delay of sexual maturation in female type. Patient has regular female external genitalia, 6 cm in length blind‐ended vagina, both testicles present in inguinal canal. The clinical phenotype of this patient was determined as CAIS. The family history of CAIS was confirmed. Pedigree anamnesis showed that mother's aunt also has AIS. Laboratory tests: FSH 4.8 IU/L (0.2‐2.8), LH 4.91 IU/L (0.1‐1.3), FT 0.0279 ng/dL (<0.04‐0.11). Mutation c.2566C>T (rs886041132) was found in patient 3 (Figure 3). The detected substitution is a missense mutation (Arg856Cys) and located in exon 7 encoding ligand binding domain. This mutation was identified previously in patients from different countries.^17^ ^,^ ^18^ ^,^ ^19^ No data are available on the allele frequency. Pathogenicity was estimated using the following scores:

- SIFT—0.001, damaging
- PolyPhen—0.994, probably damaging
- MutationTaster—1, disease causing.

For this reason, we predict this variant as pathogenic.

### 3.4 Patient 4

**Figure 4:**
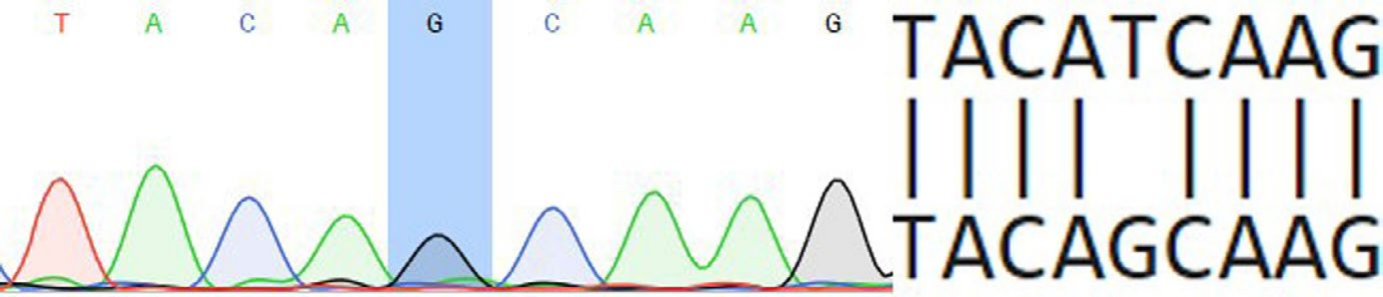
Partial electropherogram of exon 7 of androgen receptor (AR) gene

**Figure 5:**
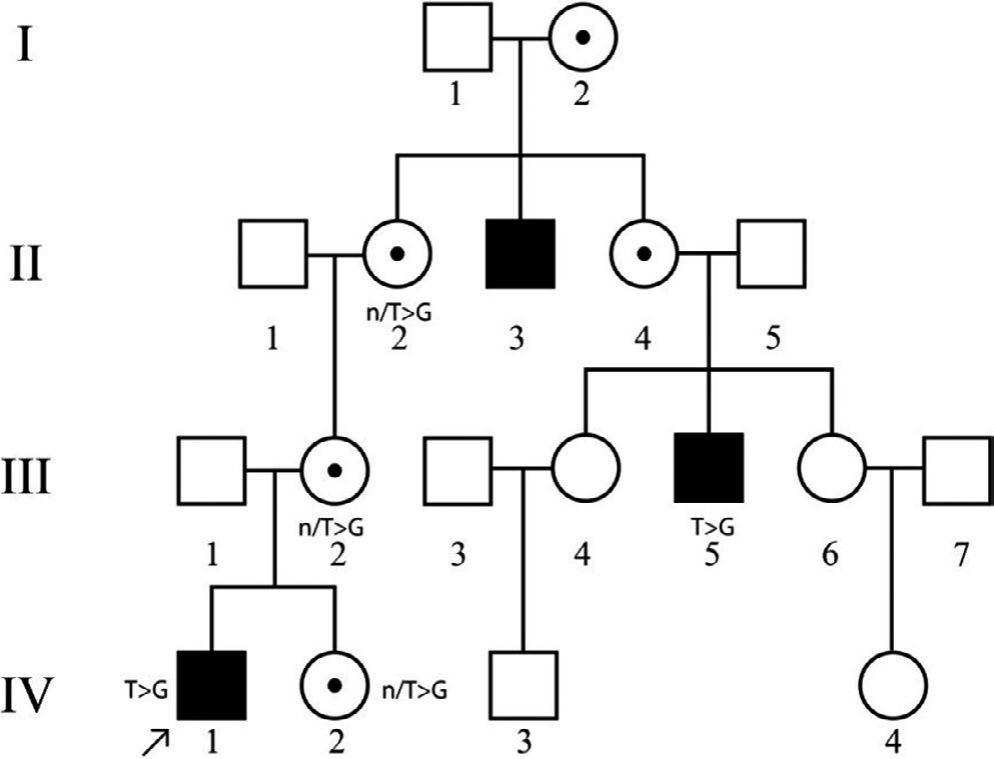
Pedigree of patient UKR1901 with identified novel c.2507T>G mutation in exon 7 of AR gene

46,XY, SRY+, was born with regular female phenotype, female external genitalia, without signs of masculinization. Patient was registered in a female social sex. At the age of 2 years and 7 months, patient was examined for bilateral “inguinal hernias”. The pedigree is burdened with AIS: Grandmother (mother's mother) has two sisters—one has AIS, and second is healthy but has AIS daughter. Ultrasonography revealed that both testicles are located in the inguinal canals. Size of the right is 28 × 9.5 × 16 mm and that of left one is 27 × 14 × 18 mm; the uterus, cervix, and both ovaries are not detected. The clinical phenotype of this patient was determined as CAIS. The family history of CAIS was confirmed. Laboratory tests: FSH 11.29 IU/L (0.2‐2.8), LH 6.68 IU/L (0.1‐1.3), TT 5.98 ng/dL (<7‐20), FT 0.0081 ng/dL (<0.04‐0.11), and dihydrotestosterone 483 pg/mL (24‐368). Mutation c.2507T>G was identified in patient 4 (Figure 4). The databases Varsome, gnomAD, and ARDB do not have data on this transversion, suggesting that this is a novel variant. The substitution was registered in ClinVar database with variation ID 974 911. This missense mutation is located in exon 7 (ligand binding domain) and results into the substitution Ile836Ser. Sequencing was also performed on family members of the proband, shown in Figure 5. It is worth noting that cousin of proband's mother (person III:5), who had also been diagnosed with CAIS, is hemizygous carrier of the same mutation. Maternal grandmother (person II:2), mother (person III:2), and healthy sibling (person IV:2, 46,XX girl) are heterozygous carriers of identified c.2507T>G mutation. Pathogenicity was evaluated using three different pathogenicity scores:

- SIFT—0, damaging
- PolyPhen—0.988, probably damaging
- MutationTaster—1, disease causing.

Obtained results allow us to consider this missense variant pathogenic.

**Figure 6:**
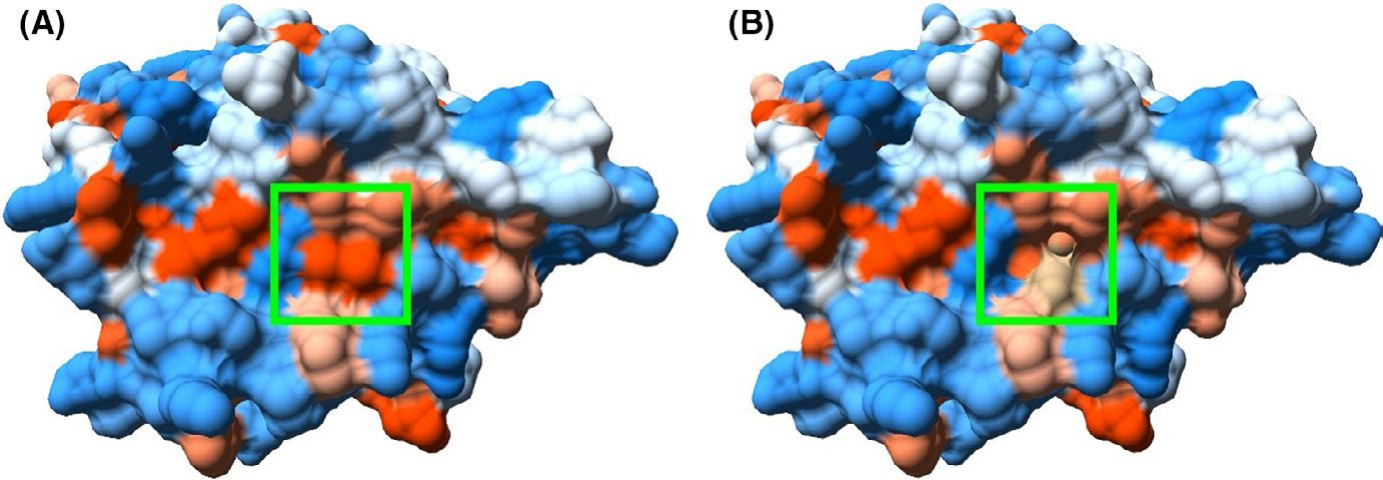
Hydrophobicity profile changes between wild‐type AR (A) and Ile836Ser mutant (B) (red—hydrophobic region, blue—hydrophilic). PDB ID: 2PIX

**Figure 7:**
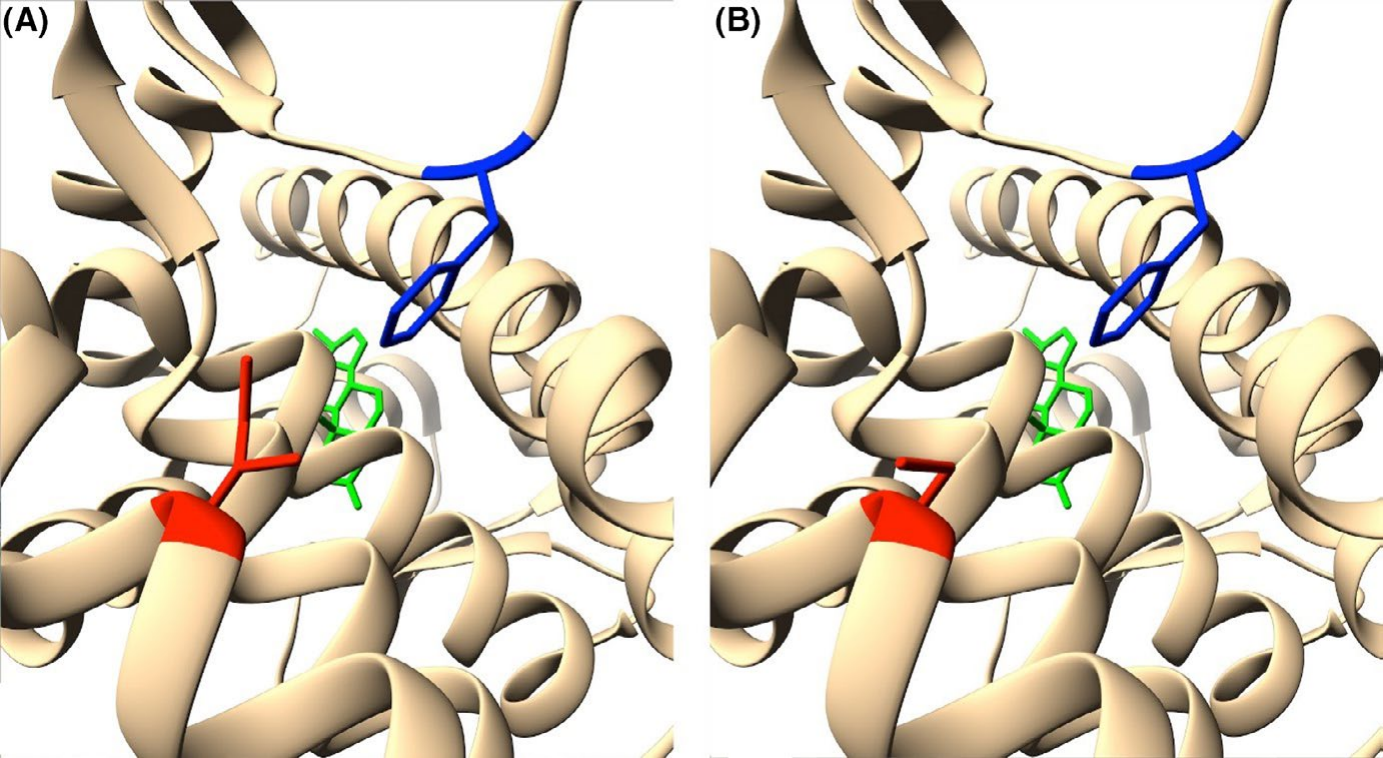
Side radical changes between wild‐type AR (A) and Ile836Ser mutant (B) (red—amino acid 836, blue—Phe916, green—DHT). PDB ID: 2PIX

Evaluation of phosphorylation pattern changes suggests that the following known AR‐specific kinases act upon mutant Ser836: PKC kinase (NetPhorest, final score—0.05); MAPK family kinases (Group‐based Prediction System, score—106 362); CDK1, CDK7, CDK9 (*P* = .01 269), kinases from the Akt (*P* = .000 912), and MAPK families (*P* = .0108) (PhosphoPICK). This amino acid substitution occurs in helix 9 of ligand binding domain, which is one of the target regions in the treatment of prostate cancer with androgen receptor antagonists. This fact and the close location of the side radical of Ile836 to Phe916 required for the binding of androgens is indicative of the pathogenicity of this mutation.^20^ Considering the substitution of amino acid radicals, it can be noted that native Isoleucine has a much longer nonpolar side chain, compared with the shorter polar serine, which also has a hydroxyl group –OH, which causes changes in protein's hydrophobicity profile (Figure 6). Predictive modeling of Ile836Ser substitution using PDB ID: 2PIX structure as a template was conducted, to measure changes in side chain distances. Analysis of the substitution on the 3D model structure showed that the distance between the side radicals Ile836 and Phe916 is 3.94 Å, while the distance between Ser836 and Phe916 is 1.58 Å more and is 5.52 Å (Figure 7). STRUM calculations of protein stability change caused by single‐point mutation showed destabilizing impact of Ile836Ser substitution. While ΔΔG < 0 result is supposed to be destabilizing, and ΔΔG <−1 considerably destabilizing, our substitution showed the result ΔΔG = −2.6. Molecular dynamics modeling is the next step in determining the nature of the pathogenicity of Ile836Ser substitution.

## 4 DISCUSSION

Three missense mutations in exon 7 and one synonymous mutation in exon 8 (both coding LBD) detected in AIS patients from Ukraine were classified as pathogenic by SIFT, PolyPhen, MutationTaster, and Human Splicing Finder, whose algorithms take into the account amino acid conservation, location of an amino acid in functionally important regions of the protein, and the existence of studied SNPs in multiple databases. Quantitative and qualitative data on ligand binding were obtained earlier for patients with c.2528T>C, c.2667C>T, c.2566C>T mutations.

In a PAIS patient (1) with c.2528T>C Ile843Thr mutation, all used resources (SIFT, PolyPhen, MutationTaster) evaluate this substitution as pathogenic. Obtained predictions correlate with described previously experimental results of Bmax (total receptor concentration in the tissue sample) −7 × 10^−12^ mol/g protein, and Kd (equilibrium dissociation constant) −0.35 × 10^−9^ M (normal Kd = 0.08 × 10^−9^ M).^21^ This argues for a reduced ability of the protein to bind androgens. Noteworthy, this mutation was found previously in a patient with CAIS phenotype. Such a difference in the severity of AIS phenotype in patients bearing the same mutation can be explained by the possible length variation in the polyG and polyQ tracts of AR.^22^

In the case of CAIS patient (3) with c.2566C>T Arg856Cys mutation all used in our study, bioinformatic resources (SIFT, PolyPhen, MutationTaster) evaluate this substitution as pathogenic. Such a prediction is in line with previously published experimental results concerning molecular consequences of such a mutation in cell culture: Bmax < 10 × 10^−18^ mol/g DNA (normal parameters in the culture of genital skin fibroblasts 630 × 10^−18^ mol/g DNA) and Kd = 0.125 × 10^−9^ M (normal value Kd = 0.08 × 10^−9^ M), which implies a reduced androgen binding ability.^23^

In our study, bioinformatic analysis of c.2667C>T Ser889 = mutation found in PAIS patient (2) using Human Splicing Finder showed introduction of a new enhancer motif for the SRp55 spliceosome protein, which results in modified mature mRNA transcript. In previous studies, this variant was described in PAIS patient and no binding of androgens was found (Bmax = 0, Kd = 0).^24^ In addition, appearance of another, significantly shorter, aberrant transcript (5.5 kb vs normal 10.5 kb) was shown, as a result of effect of this substitution on splicing.^25^ It is important that it is not a unique example of splicing perturbation by a silent mutation impact in AR gene. In 2017, another synonymous mutation in exon 1 (N‐terminal domain) that resulted into CAIS was found in two unrelated patients from Brazil.^26^

A novel missense mutation c.2507T>G in LBD of AR gene was identified in Ukrainian patient 4 with a family history of CAIS. The analysis of this variant suggests several possible mechanisms of its pathogenicity. Firstly, we determined this mutation as pathogenic using SIFT, PolyPhen, MutationTaster. It is important to note that the replacement of longer nonpolar side chain of Isoleucine by shorter polar side chain of serine destabilizes the protein, as was shown by STRUM—instrument, assessing protein stability changes, previously successfully used in assessment of free energy change in mutants with known pathogenicity.^27^ 3D modeling of Ile836Ser mutant protein was performed based on a protein crystal structure. The changes in hydrophobicity profile (Figure 6) and enlarged distance between amino acid residue substitution 836 and Phe916 (Figure 7) are shown. Previously, it was identified that Phe916 is crucial for androgen binding. It was shown that Phe916Ala mutant binds the androgens 460 times worse than wild‐type protein.^20^ Moreover, AR is known to undergo post‐translational phosphorylation (before and after ligand binding), which affects the protein functions depending on the site of phosphorylation.^6^ ^,^ ^7^ ^,^ ^8^ In our study, we have predicted the aberrant phosphorylation of mutant Ser836 by kinases from MAPK and Akt families and by kinases CDK1, CDK7, CDK9, and PKC. Androgens are known to rapidly activate kinase signaling cascade and modulate intracellular level of calcium ions. Binding of dihydrotestosterone to AR allows the receptor to interact and activate the Src tyrosine kinase. In turn, the activation of Src kinase leads to the phosphorylation of EGFR (Epidermal growth factor receptor). It has also been shown that AR is rapidly (within 5 minutes) phosphorylated after the interaction with dihydrotestosterone, which leads to the activation of ERK and CREB kinases within 1 minute. This is necessary to maintain spermatogenesis in Sertoli cells. It was shown that it takes dihydrotestosterone at least 45 minutes to induce transcriptional activity in addition to time required for protein synthesis. Thus, the appearance of a new phosphorylation site, which was predicted in our study by NetPhorest 2.1, Group‐based Prediction System 5.0, and PhosphoPICK resources, may affect functions of AR.

## 5 CONCLUSION

A novel missense mutation, c.2507T>G, in LBD of AR gene was identified in a Ukrainian patient with a family history of CAIS (see Figure 3) and predicted as pathogenic using bioinformatic tools and analysis of 3D model of Ile836Ser mutant protein structure. Results concerning the pathogenicity of c.2528T>C (rs9332970), c.2566C>T (rs886041132), and c.2667C>T (rs137852594) mutations detected in patients with AIS from Ukraine obtained using bioinformatic resources SIFT, PolyPhen, MutationTaster, and Human Splicing Finder correlate with previously published data concerning weaker binding of androgens in patients with the same mutations. This approves informativity of using such resources for mutation pathogenicity analysis.

## CONFLICT OF INTEREST

The authors have no conflicts of interest to declare.

## AUTHOR CONTRIBUTIONS

D. S.: performed bioinformatic analysis of molecular data and contributed to writing the manuscript. O. G.: conducted molecular analysis of AR gene exon sequences. D. L.: performed modeling and analysis of mutant protein 3D structure. G. L.: collected clinical data and analyzed cytogenetic data. N. Z.: supervised clinical investigation of patients and family members. L. L.: designed and supervised the molecular analysis and prepared the manuscript.

## ETHICAL APPROVAL

Ethical approval of this study was obtained from the committee on bioethics of the Institute of Molecular Biology and Genetics of National Academy of Sciences of Ukraine, protocol No. 2 (30.04.2013). Informed consent was obtained from all patients and/or their parents.

## Data Availability

The data that support the findings of this study are available from the corresponding author upon reasonable request.
